# Diagnostic Performance of Fractional Flow Reserve From CT Coronary Angiography With Analytical Method

**DOI:** 10.3389/fcvm.2021.739633

**Published:** 2021-10-20

**Authors:** Jun-Mei Zhang, Huan Han, Ru-San Tan, Ping Chai, Jiang Ming Fam, Lynette Teo, Chee Yang Chin, Ching Ching Ong, Ris Low, Gaurav Chandola, Shuang Leng, Weimin Huang, John C. Allen, Lohendran Baskaran, Ghassan S. Kassab, Adrian Fatt Hoe Low, Mark Yan-Yee Chan, Koo Hui Chan, Poay Huan Loh, Aaron Sung Lung Wong, Swee Yaw Tan, Terrance Chua, Soo Teik Lim, Liang Zhong

**Affiliations:** ^1^National Heart Centre Singapore, Singapore, Singapore; ^2^Duke-NUS Medical School, Singapore, Singapore; ^3^Department of Cardiology, National University Heart Centre, Singapore, Singapore; ^4^Yong Loo Lin School of Medicine, National University of Singapore, Singapore, Singapore; ^5^Department of Diagnostic Imaging, National University Hospital, Singapore, Singapore; ^6^Institute for Infocomm Research, Agency for Science, Technology and Research, Singapore, Singapore; ^7^California Medical Innovations Institute, San Diego, CA, United States

**Keywords:** coronary artery disease, fractional flow reserve, computed tomography coronary angiography, analytical method, non-invasive

## Abstract

The aim of this study was to evaluate a new analytical method for calculating non-invasive fractional flow reserve (FFR_AM_) to diagnose ischemic coronary lesions. Patients with suspected or known coronary artery disease (CAD) who underwent computed tomography coronary angiography (CTCA) and invasive coronary angiography (ICA) with FFR measurements from two sites were prospectively recruited. Obstructive CAD was defined as diameter stenosis (DS) ≥50% on CTCA or ICA. FFR_AM_ was derived from CTCA images and anatomical features using analytical method and was compared with computational fluid dynamics (CFD)-based FFR (FFR_B_) and invasive ICA-based FFR. FFR_AM_, FFR_B_, and invasive FFR ≤ 0.80 defined ischemia. A total of 108 participants (mean age 60, range: 30–83 years, 75% men) with 169 stenosed coronary arteries were analyzed. The per-vessel accuracy, sensitivity, specificity, and positive predictive and negative predictive values were, respectively, 81, 75, 86, 81, and 82% for FFR_AM_ and 87, 88, 86, 83, and 90% for FFR_B_. The area under the receiver operating characteristics curve for FFR_AM_ (0.89 and 0.87) and FFR_B_ (0.90 and 0.86) were higher than both CTCA- and ICA-derived DS (all *p* < 0.0001) on per-vessel and per-patient bases for discriminating ischemic lesions. The computational time for FFR_AM_ was much shorter than FFR_B_ (2.2 ± 0.9 min *vs*. 48 ± 36 min, excluding image acquisition and segmentation). FFR_AM_ calculated from a novel and expeditious non-CFD approach possesses a comparable diagnostic performance to CFD-derived FFR_B_, with a significantly shorter computational time.

## Introduction

Atherosclerotic plaque deposition in the coronary arterial wall results in anatomical stenosis that may reduce perfusion and induce ischemia in the subtended myocardial territory (1). Fractional flow reserve (FFR), measured during invasive coronary angiography (ICA), is the reference standard for quantifying the functional significance of coronary artery stenoses and discriminating ischemic lesions (2, 3). However, ICA-based FFR measurement incurs additional resource utilization, increases procedural time, and is associated with greater patient discomfort (4). Recently, non-invasive FFR (FFR_CT_) derived from computed tomography coronary angiography (CTCA) images and computational fluid dynamics (CFD) has demonstrated feasibility for the identification of ischemic coronary lesions (5) with reasonable diagnostic accuracy (6) and prognostication (7).

Mesh generation and iterative solution of numerical equations integral to CFD demand long computational time for the calculation of time-varying instantaneous values of coronary blood flow parameters like velocity, pressure, *etc*. The current CFD-based FFR_CT_ methods take 1 to 4 h per FFR_CT_ analysis (8). Reduced-order (9–11), steady-flow (12) CFD simulations and predictive models using machine learning (13–15) may improve computational efficiency and facilitate shorter turnaround times and/or on-site analysis, which will help garner a wider adoption of non-invasive FFR.

Still an analytical method to calculate FFR non-invasively without the need for computationally demanding CFD modeling would further simplify the derivation of non-invasive FFR from CTCA images. Huo et al. (16) proposed an analytical model that embodied integral equations to be solved based on the dimensions of anatomical stenosis on CTCA and estimates of hyperemic coronary flow derived from *in vitro* and *in vivo* animal experiments. In this study, we developed an original analytical method, FFR_AM_, that relies on neither CFD nor other inputs other than CTCA images. Flow rate through coronary lesions (*Q*_AM_) was estimated from anatomical data reconstructed from CTCA, where anatomical features known to influence the hemodynamics in stenotic arteries, including lesion length, lumen area, flow entrance, and exit angles (17), were explicitly considered. Our aim is to assess the diagnostic performance of FFR_AM_ with reference to our previously developed CFD-based FFR_B_ and invasive FFR in a cohort of coronary artery disease (CAD) patients.

## Materials and Methods

### Study Design and Study Population

The current study consecutively enrolled patients from two tertiary centers, with age ≥ 21 years, who had undergone CTCA, and were scheduled to undergo clinically indicated ICA and FFR measurement. The time difference between CTCA and ICA was 32 (19–51) days (median, interquartile range). The exclusion criteria included prior coronary revascularization, acute coronary syndrome occurring between 30 days before CTCA and ICA, angina at rest, left ventricular ejection fraction <30%, hypertrophic cardiomyopathy, significant valve disease including prosthetic heart valve, implanted pacemaker or defibrillator, complex congenital heart disease, estimated glomerular filtration rate <30 ml/min/1.73 m^2^, tachycardia or significant arrhythmia, iodinated contrast allergy, contraindication to beta-blocker, nitroglycerin, or adenosine, serious comorbidity with life expectancy <2 years, and pregnancy. The study was approved by the local institutional review boards, and all participants gave written informed consent.

**Figure 1:**
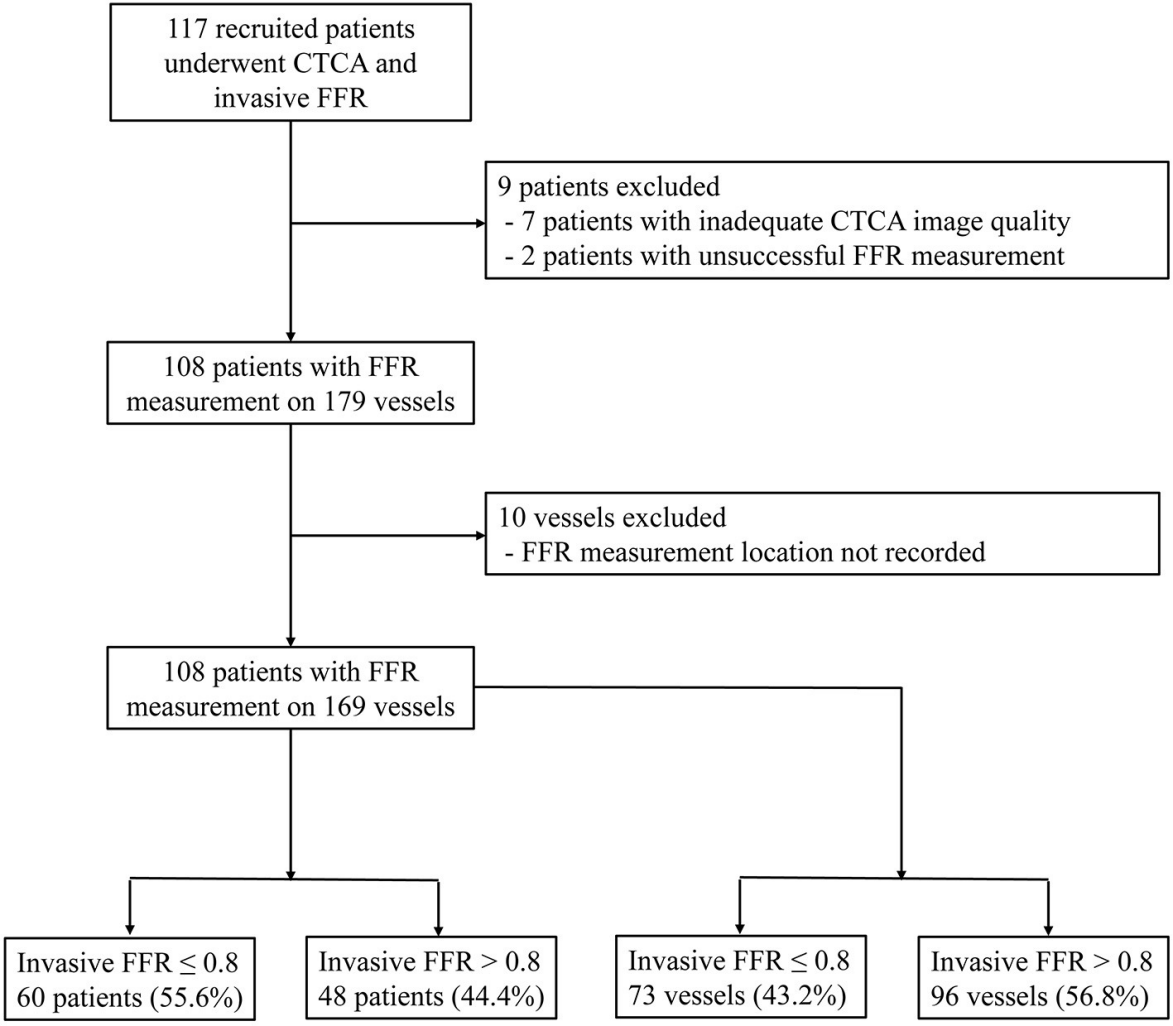
Study inclusion flowchart. CTCA, CT coronary angiography; FFR, fractional flow reserve.

From September 20, 2016 to March 25, 2020, 117 participants were recruited. Nine subjects were excluded: two patients with unsuccessful invasive FFR measurement and seven patients with inadequate CTCA image quality. Among the seven patients, one patient had blooming artifacts due to extreme coronary calcification (Agatston score 3441), and six patients had motion artifacts in the CTCA images. By excluding 10 vessels with missing video recordings of the FFR measurement locations, 108 participants with 169 vessels were included in the analysis (Figure 1).

### ICA and FFR Measurement

For the recruited patients, invasive FFR measurement was performed according to the institutional protocol. Every participant underwent ICA *via* either the femoral or radial approach using 5F, 6F, or 7F diagnostic or guiding catheters (18). Angiography was performed in standard projections. Diameter stenosis at ICA (DS_ICA_) was visually assessed (19), and lesions were deemed obstructive if DS_ICA_ ≥50%. The pressure wires/catheters used for the invasive FFR can be found in the Supplementary Material. Intra-coronary pressure was measured at the ascending aorta and distal to the coronary lesion in at least one vessel. Hyperemia was induced by either intravenous infusion (140–180 μg/kg/min) or an intracoronary bolus (60–200 μg) of adenosine. A coronary lesion was categorized as ischemic if FFR ≤ 0.80. Two consultant interventional cardiologists with extensive clinical experience reviewed the ICA images, and the lesions were evaluated based on overall consensus. In case of disagreement, a third independent cardiologist reviewed the films and provided a final diagnosis.

### CTCA Acquisition

Every participant underwent CTCA on one of the following scanners with ≥256 detector rows: Toshiba Aquilion One 320 Slice, Canon Aquilion ONE Genesis 640 Slice, Philips Brilliance iCT 256-detector, Siemens Somatom Force dual source 384-detector, GE Revolution single source, and Siemens Somatom Drive dual source 256-detector. Oral beta-blocker (metoprolol) was administered to the participants with a heart rate >65 beats per min (20). Sublingual glyceryl trinitrate was administered just prior to scanning for optimal coronary vasodilation during image acquisition. Prospective electrocardiogram-triggered protocol was used to acquire image data at pre-specified phases of the heart cycle, and CTCA scan was performed at inspiratory breath-hold. Then, 50 to 75 ml of non-ionic contrast Omnipaque 350 was administered for each scan.

The CTCA studies were read by an accredited reporting radiologist or cardiologist and verified by a second accredited reader. The diameter stenoses of coronary lesions on CTCA images (DS_CTCA_) were graded according to anatomical severity: normal, absent plaque, and no luminal stenosis; minimal, DS_CTCA_ <25%; mild, 25% ≤ DS_CTCA_ ≤ 49%; moderate, 50% ≤ DS_CTCA_ ≤ 69%; severe, 70% ≤ DS_CTCA_ ≤ 99%; and occluded, DS_CTCA_ = 100% (20). A coronary lesion was deemed obstructive if DS_CTCA_ ≥50%.

### CTCA Image Segmentation and 3D Model Reconstruction

**Figure 2:**
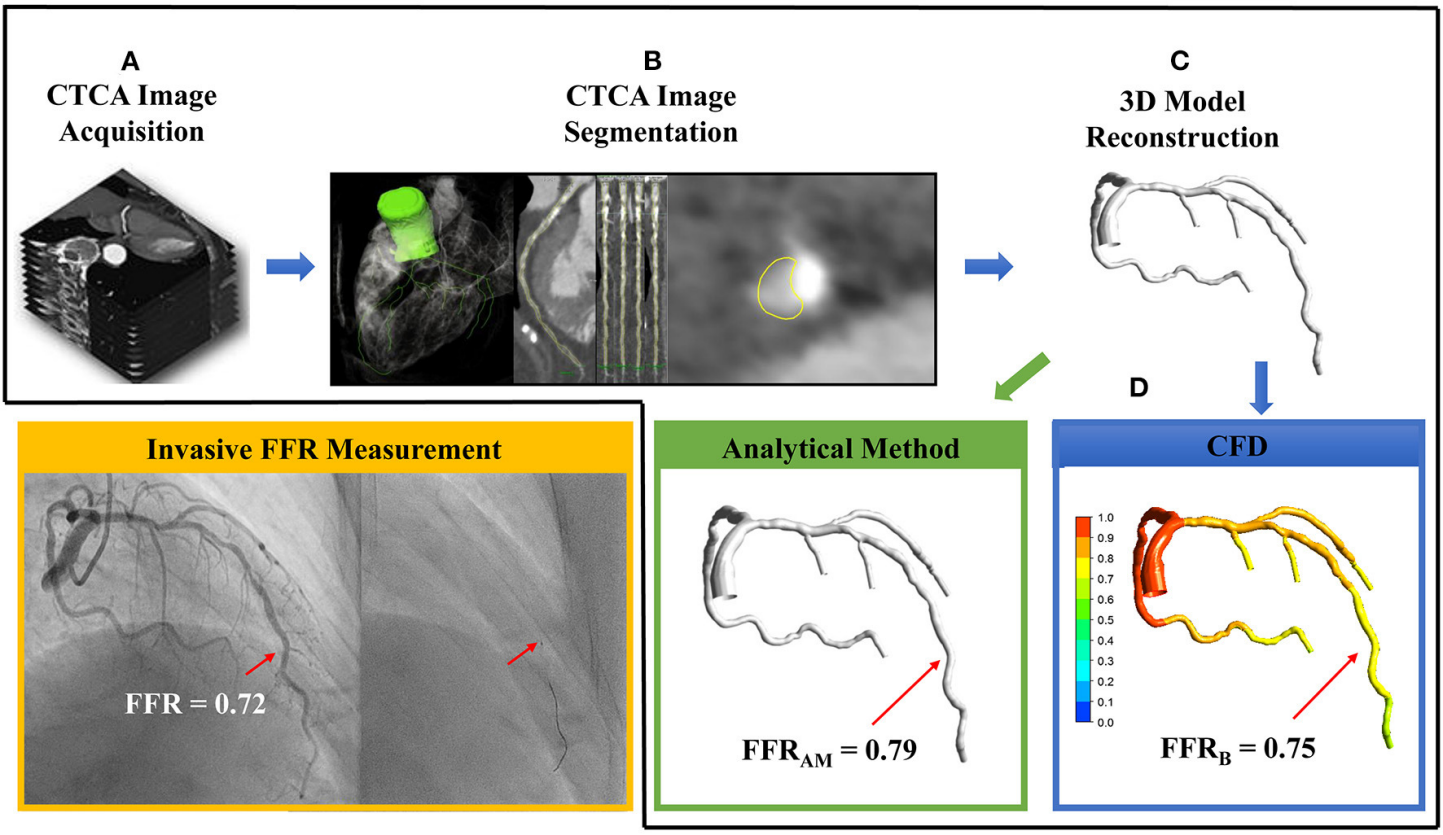
The sequential steps involved in the calculation of non-invasive fractional flow reserve (FFR) include **(A)** computed tomography coronary angiography (CTCA) image acquisition, **(B)** CTCA image segmentation *via* extracting centerlines and delineating lumen contours in the transversal and cross-sectional images, **(C)** 3D reconstruction of subject-specific coronary artery tree, and **(D)** using either analytical method to calculate FFR_AM_ (green box) or computational fluid dynamics simulation to compute FFR_B_ (blue box). In this case, FFR_AM_ and FFR_B_ were 0.79 and 0.75, respectively, at the site of invasive FFR 0.72 measured with a pressure catheter (right, inset) during invasive coronary angiography (yellow box).

**Figure 3:**
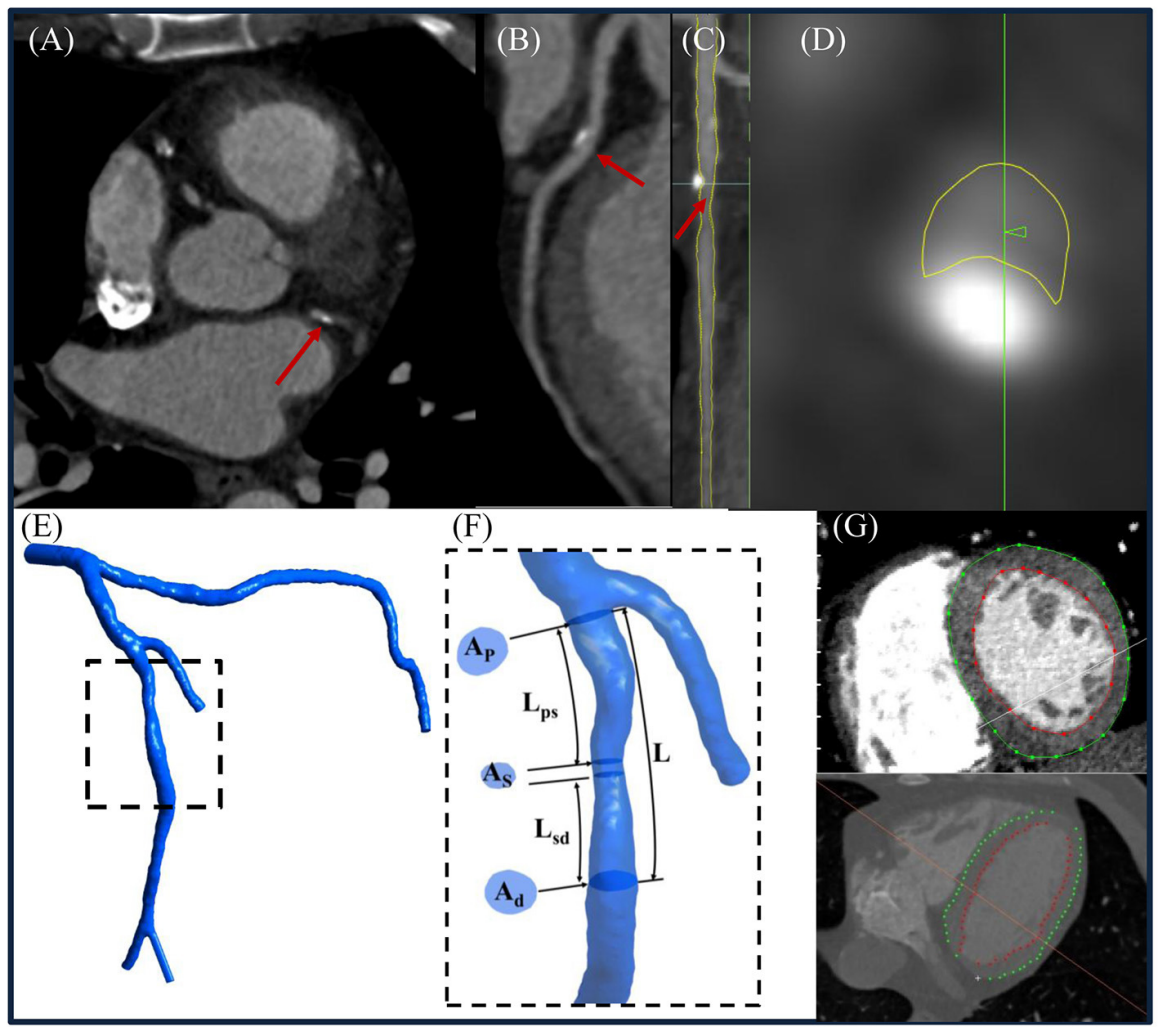
**(A)** Representative computed tomography coronary angiography (CTCA) cross-sectional slice, **(B)** curved multiplanar reconstructed images of a left anterior descending (LAD) coronary artery, **(C)** straightened LAD with segmented lumen lines (yellow color) in transversal and **(D)** cross-sectional views, **(E)** reconstructed 3D left coronary tree, and **(F)** enlarged view to illustrate how to measure *A*_p_, *A*_s_ , *A*_d_, *L*_ps_, *L*_sd_, and *L* from the model. **(G)** Left ventricular (LV) endocardial and epicardial contours were delineated from CTCA images to calculate the LV mass. (Note: *A*_p_, *A*_s_, and *A*_d_ represented the lumen area at proximal, maximally stenosed, and distal segments, respectively; *L*_ps_, *L*_sd_, and *L* were the length measured from the proximal end of the coronary lesion to the proximal end of the maximally stenosed segment, the distal end of the maximally stenosed segment to the distal end of the coronary lesion, and the proximal to the distal ends of the whole coronary lesion, respectively. Flow entrance and exit angles (α and β) were then calculated using Eqs. (A-1) and (A-6) in the Supplementary Material, respectively.

Dedicated QAngio CT software (21) (version 3.0, Medis) was used for segmentation and 3D reconstruction of coronary artery. Additional details are found in the Supplementary Material. The surface meshes of the 3D coronary artery tree model were generated using 3D Workbench (version 0.8, Medis). Figure 2 illustrates the workflow for non-invasive FFR calculation in a participant. Figure 3 depicts the detailed coronary anatomy in another participant with pertinent anatomical parameter inputs for calculating the FFR_AM_.

Total coronary flow under resting conditions, a required input parameter for non-invasive FFR estimation, is linearly related to left ventricular mass (LVM) (22). The latter was measured using validated Segment CT software (version 2.2, Medviso) (23) that semi-automatically delineated left ventricular (LV) endocardial and epicardial contours on contiguous 2D LV short-axis slices reformatted from the CTCA-reconstructed 3D whole-heart model (Figure 3).

### Computation of Non-invasive FFR_AM_ With Analytical Model

In our analytical model, $\text{FF}{\text{R}}_{\text{AM}}=1-\frac{P_{1}+P_{2}}{P_{a}}$, where *P*_a_ is patient-specific mean aortic pressure estimated as mean cuff pressure minus 6.8 mmHg to account for pressure drop during hyperemia (24), and Δ*P*_1_ and Δ*P*_2_ are pressure drops across the coronary lesion and from the coronary orifice to the proximal end of the coronary lesion, respectively. The latter is calculated from the Hagen–Poiseuille equation according to the viscosity of the blood, lumen area, length, and flow rate of each coronary branch (from the coronary orifice to the proximal end of the coronary lesion), respectively.

By law of energy conservation, Δ*P*_1_ entails convective and diffusive energy losses as well as energy loss attributable to sudden constriction and expansion (16). Flow separation and swirling that exacerbate energy losses and pressure drops are related to features such as lesion length, lumen area, flow entrance, exit angles, *etc*. (25). We applied these considerations in series to a coronary lesion model of total length *L* decomposed schematically into three components: a proximal contracting segment of length *L*_ps_ and distal expanding segment of length *L*_sd_, which bookend a middle maximally stenosed segment of finite length *L*–*L*_ps_-*L*_sd_ (Supplementary Figure 1). The respective pressure drops across the three segments Δ*P*_ps_, Δ*P*_sd_, and Δ*P*_ss_ sum up to Δ*P*_1_ and are, from a mechanical engineering perspective, analogous to pressure drops across contracting, expanding, and straight pipes, respectively (Supplementary Material). Figure 3F illustrates how we measured the anatomical parameters *L, L*_ps_, and *L*_sd_ as well as *A*_P_, *A*_d_, and *A*_s_, the lumen areas at the proximal and distal ends of the coronary lesion, and the maximally stenosed segment, respectively. From these parameters, flow entrance (α) and exit (β) angles were derived to facilitate the calculation of Δ*P*_ps_ and Δ*P*_sd_ (Supplementary Material).

To calculate the hyperemic flow rate of each coronary branch, we first calculated the total coronary flow rate at resting from CTCA-assessed LVM (22) and then estimated the resting flow rate through the *i*-th coronary branch using the scaling law (26). Finally, hyperemic flow rate through a coronary lesion located at the *i*-th branch of the coronary artery tree was computed as *k* times of its value at resting state_._ (24). The coefficient *k* reflects the magnitude of flow increase at hyperemia and is dependent on the diameter stenosis of the lesion (DS). Inputting *Q*_AM_ to the analytical model, Δ*P*_1_ and then FFR_AM_ could be calculated without a need for CFD simulation (Supplementary Material).

### Computation of Non-invasive FFR_B_ Based on Reduced-Order CFD Simulation

Reduced-order CFD simulation was performed on the reconstructed 3D coronary artery tree model in deriving non-invasive FFR_B_ measurement. Additional details can be found in our prior studies (9, 11, 27) and in the Supplementary Material. The FFR_B_ value was extracted at the location on the 3D coronary tree model that best corresponded to the site of the FFR measurement at ICA as judged by cardiologists (JMF and CYC).

### Statistical Analysis

Continuous variables were summarized as mean ± standard deviation (SD) or median (interquartile range), and the categorical variables were summarized as frequencies and percentages. Two-sample *t*-test, Wilcoxon rank-sum test, and Fisher's exact test were used to compare the ischemic and non-ischemic groups on continuous normally distributed variables, continuous parameters with non-normal distribution, and binary variables, respectively. For vessels with multiple lesions, the pressure drops over individual lesions were compared, and the anatomical parameters associated with the lesion contributing to the largest pressure drop were selected for statistical analysis. The DeLong test (28) was used to compare receiver operating characteristic (ROC) areas under the curve (AUCs). Accuracy, sensitivity, specificity, positive prediction value, negative predictive value (NPV), and likelihood ratios corresponding to the diagnostic threshold were calculated to enable a comparison of the discrimination capability among DS_CTCA_, DS_ICA_, and non-invasive FFR indexes. SPSS (version 22, IBM, New York, USA) was used to perform the statistical analyses. Statistical significance was set at *p* <0.05.

## Results

### Patient Characteristics

Detailed demographics of the 108 participants (mean age 60 ± 9 years; 81 males) is presented in Table 1. Ethnicities included Chinese (80%), Indian/Malay (15%), and other Asians (5%) which closely reflect the ethnic percentages of the Singapore population. The majority of the participants had hypertension (64%) and hyperlipidemia (70%).

**Table 1:** Patient characteristics.

	**Mean ± SD, median (interquartile), or *n* (%)**
Age, years	60 ± 9
Male	81 (75)
BMI, kg/m^2^	26.1 ± 4.8
Heart rate at CTCA, bpm	57 ± 6
**Race/ethnics**	
Chinese, *n* (%)	86 (80)
Indian/Malay, *n* (%)	16 (15)
Other Asians, *n* (%)	6 (5)
**Risk factors**	
Hypertension, *n* (%)	69 (64)
Hyperlipidemia, *n* (%)	76 (70)
Diabetes, *n* (%)	30 (28)
Current smoker, *n* (%)	16 (15)
Ex-smoker, *n* (%)	9 (8)
**Vital signs**	
SBP, mmHg	134 ± 17
DBP, mmHg	77 ± 11
**Laboratory measures**	
Hemoglobin, g/dl	13.9 ± 1.3
Hematocrit, %	41.9 ± 3.4
Creatinine, mmol/L	0.076 ± 0.019
**Medications**	
Aspirin, *n* (%)	94 (87)
Beta-blocker, *n* (%)	54 (50)
Nitrate, *n* (%)	72 (67)
Statins, *n* (%)	91 (84)
ACEI/ARB, *n* (%)	31 (29)
Clopidogrel, *n* (%)	93 (86)
Calcium channel blockers, *n* (%)	23 (21)
Other medications, *n* (%)	52 (48)
Left ventricular mass, g	115 ± 31
Agatston score	275 (108, 502)

*BMI, body mass index; CTCA, computed tomography coronary angiography; SBP, systolic blood pressure; DBP, diastolic blood pressure; ACEI, angiotensin-converting enzyme injection; ARB, angiotensin receptor blocker*.

### Characteristics of Flow Rate and Morphological Parameters

**Figure 4:**
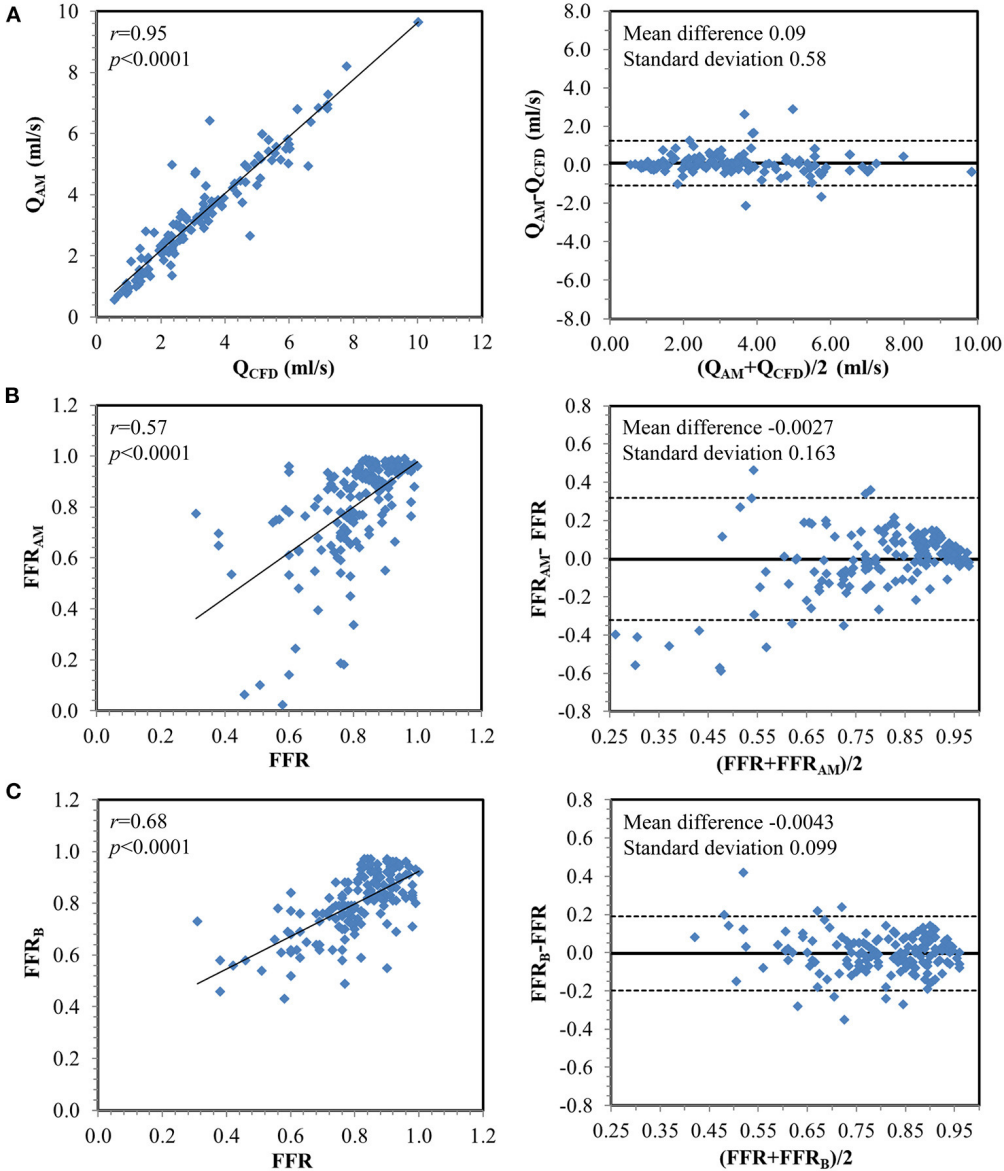
Correlation and Bland–Altman plots **(A)** between flow rates calculated from computational fluid dynamics simulation (*Q*_CFD_) and estimated from the analytical model (*Q*_AM_) and between invasively measured fractional flow reserve (FFR) with **(B)** FFR_AM_ and **(C)** FFR_B_ on a per-vessel basis.

Among 169 vessels, 73 (43%) were ischemic (Table 2). *A*_s_ and *A*_d_ were significantly smaller, and α and β were significantly greater among ischemic vs. non-ischemic lesions, which contribute to the significantly greater Δ*P*_ps_ and Δ*P*_sd_ along the contracting and expanding segments, respectively, in the ischemic lesions. There was excellent correlation between flow rates through lesions derived using empirical equations and CFD simulation (mean *Q*_AM_ 3.38 ± 1.93 ml/s vs. mean *Q*_CFD_ 3.30 ± 1.97 ml/s; *r* = 0.95, *p* < 0.0001) (Figure 4).

**Table 2:** Characteristics of flow rate, anatomical parameters, and pressure drop over various coronary lesion segments to calculate the non-invasive FFR_AM_ overall and by study group (ischemic group: FFR ≤ 0.8; non-ischemic group: FFR > 0.8).

**Parameter**	**Overall** **(*n* = 169)**	**FFR > 0.80** **(*n* = 96)**	**FFR ≤ 0.8** **(*n* = 73)**	***p*-value**
*Q*_CFD_ (ml/s)	3.30 ± 1.97	3.27 ± 2.23	3.33 ± 1.67	0.855
*Q*_AM_ (ml/s)	3.38 ± 1.93	3.21 ± 2.17	3.55 ± 1.67	0.295
*A*_p_ (mm^2^)	6.80 ± 3.66	7.58 ± 3.79	6.03 ± 3.38	0.012
*A*_s_ (mm^2^)	3.80 ± 2.16	4.56 ± 2.41	3.05 ± 1.56	<0.0001
*A*_d_ (mm^2^)	6.62 ± 3.22	7.35 ± 3.47	5.90 ± 2.79	0.008
*L* (mm)	10.77 ± 6.74	10.07 ± 5.97	11.46 ± 7.40	0.223
*L*_ps_ (mm)	3.71 ± 3.24	3.64 ± 3.44	3.78 ± 3.06	0.800
*L*_sd_ (mm)	3.51 ± 2.79	3.31 ± 2.56	3.70 ± 3.01	0.420
α (°)	9.35 ± 9.16	7.15 ± 8.49	12.28 ± 9.25	<0.0001
β (°)	10.06 ± 9.23	7.54 ± 8.07	13.39 ± 9.66	<0.0001
*P*_*ps*_ (*mmHg*)	3.37 ± 4.08	1.76 ± 2.71	4.95 ± 4.57	<0.0001
*P*_*sd*_ (mmHg)	7.10 ± 11.09	2.92 ± 4.63	11.23 ± 13.79	<0.0001
*P*_*ss*_ (mmHg)	1.03 ± 1.64	0.48 ± 0.60	1.59 ± 2.11	<0.0001

*Q_CFD_, flow rate derived from computational fluid dynamics (CFD); Q_AM_, flow rate estimated from analytical model (AM); A_p_, lumen area at the proximal end of the coronary lesions; A_s_, lumen area at the maximally stenosed segment; A_d_, lumen area at the distal end of the coronary lesions; L, lesion length; L_ps_, length of the segment from the proximal end of the coronary lesion to the proximal end of the maximally stenosed segment; L_sd_, length of the segment from the distal end of the maximally stenosed segment to the distal end of the coronary lesion; α, flow entrance angle at the distal end of the proximal contracting segment; β, flow exit angle at the proximal end of the distal expanding segment; P_ps_, pressure drop due to the contraction of the lumen area at the proximal contracting segment; P_sd_, pressure drop due to the expansion of lumen area at the distal expanding segment; P_ss_, pressure drop along the straight maximally stenosed segment*.

### Diagnostic Performance of FFR_AM_ for Discriminating Ischemic Lesions

**Figure 5:**
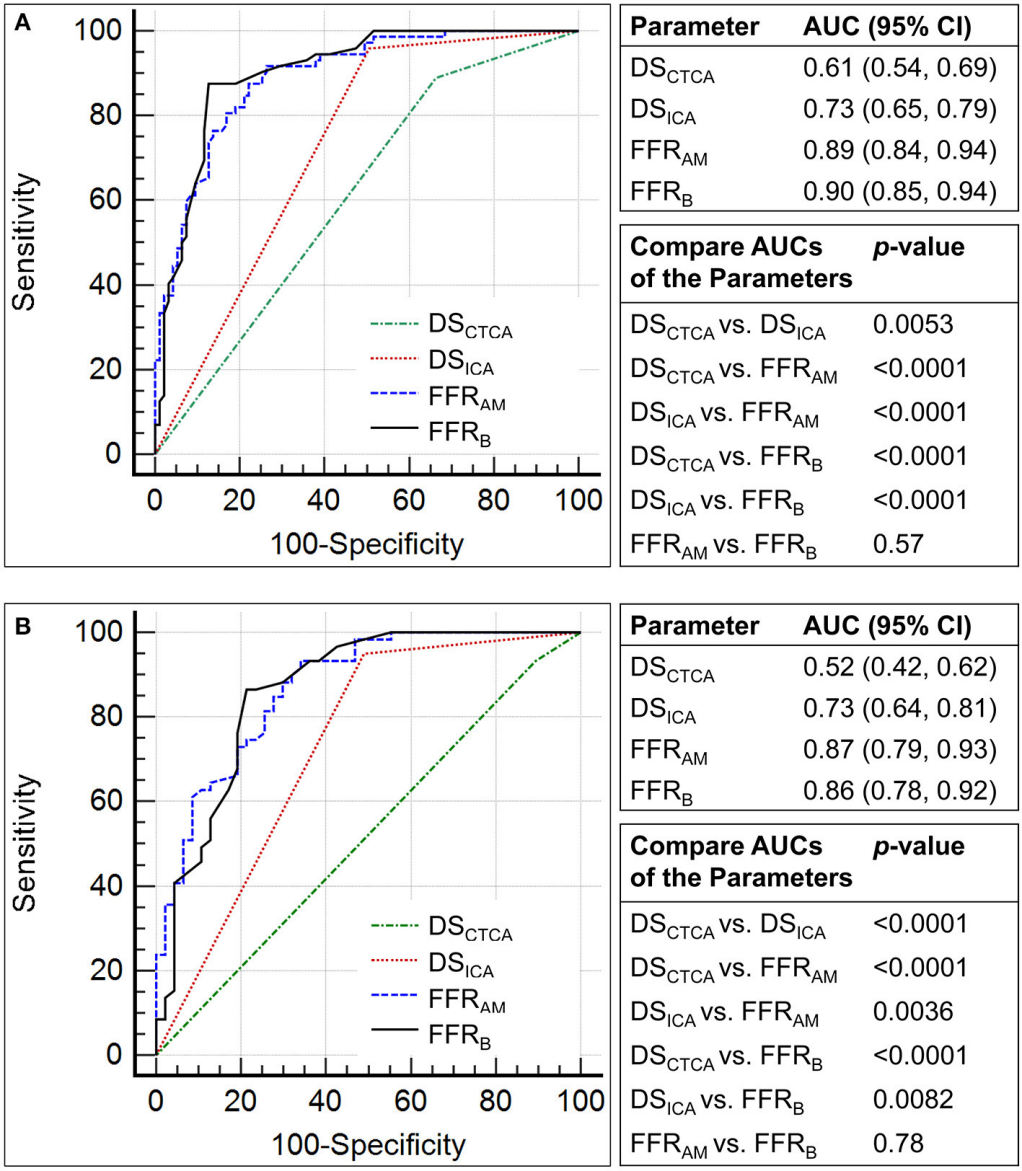
Comparison of the receiver operating characteristic curves for the discrimination of myocardial ischemia (fractional flow reserve, FFR ≤0.8) using diameter stenosis (DS; DS_CTCA_ and DS_ICA_, with a threshold of 50%) and non-invasive FFR (FFR_AM_ and FFR_B_) on **(A)** per-vessel and **(B)** per-patient levels.

Compared with invasive FFR (mean 0.81 ± 0.13), FFR_AM_ (mean 0.80 ± 0.20) exhibited fair correlation (*r* = 0.57, *p* < 0.0001) and agreement with small systematic biases (-0.0027 ± 0.163) (Figure 4). Performance metrics using DS_CTCA_ ≥50%, DS_ICA_ ≥50%, FFR_AM_ ≤ 0.8, and FFR_B_ ≤ 0.8 to discriminate ischemic lesions are compared in Table 3 and Figure 5. On a per-vessel level, the ROC AUCs (95% CI) for FFR_AM_ [0.89 (0.84, 0.94)] and FFR_B_ [0.90 (0.85, 0.94)] were significantly higher than those for DS_CTCA_ [0.61 (0.54, 0.69)] and DS_ICA_ [0.73 (0.65, 0.79)]. On a per-patient level, the ROC AUCs (95% CI) for FFR_AM_ [0.87 (0.79, 0.93)] and FFR_B_ [0.86 (0.78, 0.92)] were significantly higher than those for DS_CTCA_ [0.52 (0.42, 0.62)] and DS_ICA_ [0.73 (0.64, 0.81)]. DS_ICA_ had a higher AUC than DS_CTCA_ (both *p* <0.05 on per-vessel and per-patient analyses). There was no significant difference between FFR_AM_ and FFR_B_ in AUCs on both per-vessel and per-patient analyses (Figure 5).

**Table 3:** **(A)** Diameter stenoses (DS_CTCA_ and DS_ICA_) and non-invasive FFR (FFR_AM_ and FFR_B_) in study groups (FFR > 0.8 and FFR ≤ 0.8; **(B)** Comparison of diagnostic performance of different parameters for predicting myocardial ischemia at per-vessel level; **(C)** Comparison of diagnostic performance of different parameters for predicting myocardial ischemia at per-patient level.

**Parameter**	**Overall (*****n*** **=** **169)**		**FFR** **>** **0.80 (*****n*** **=** **96)**		**FFR** **≤** **0.8 (*****n*** **=** **73)**		***p-*value**
**(A)**							
DS_CTCA_ ≥ 50%	129 (76%)		64 (67%)		65 (89%)		0.001
DS_ICA_ ≥5 0%	119 (70%)		49 (51%)		70 (96%)		<0.0001
DS_CTCA_ ≥70%	54 (32%)		35 (36%)		19 (26%)		<0.0001
DS_ICA_ ≥70%	59 (35%)		44 (46%)		15 (21%)		<0.0001
FFR_AM_	0.80 ± 0.20		0.91 ± 0.09		0.67 ± 0.22		<0.0001
FFR_B_	0.80 ± 0.12		0.87 ± 0.08		0.71 ± 0.10		<0.0001
**Threshold**	**Accuracy**	**Sens**	**Spec**	**LR+**	**LR-**	**PPV**	**NPV**
**(B)**							
DS_CTCA_ ≥ 50%	0.57	0.89	0.33	1.33	0.33	0.50	0.80
DS_ICA_ ≥ 50%	0.69	0.96	0.49	1.88	0.09	0.59	0.94
DS_CTCA_ ≥ 70%	0.66	0.47	0.81	2.49	0.65	0.65	0.67
DS_ICA_ ≥ 70%	0.76	0.63	0.86	4.57	0.43	0.78	0.75
FFR_AM_ ≤ 0.8	0.81	0.75	0.86	5.48	0.29	0.81	0.82
FFR_B_ ≤ 0.8	0.87	0.88	0.86	6.39	0.14	0.83	0.90
**(C)**							
DS_CTCA_ ≥ 50%	0.57	0.93	0.11	1.04	0.63	0.57	0.56
DS_ICA_ ≥ 50%	0.75	0.95	0.51	1.94	0.10	0.71	0.89
DS_CTCA_ ≥ 70%	0.59	0.49	0.72	1.78	0.70	0.69	0.53
DS_ICA_ ≥ 70%	0.69	0.63	0.77	2.68	0.49	0.77	0.62
FFR_AM_ ≤ 0.8	0.75	0.73	0.79	3.42	0.34	0.81	0.70
FFR_B_ ≤ 0.8	0.82	0.86	0.77	3.69	0.18	0.82	0.82

*Sen, sensitivity; Spec, specificity; LR+, positive likelihood ratio; LR-, negative likelihood ratio; PPV, positivity predictive value; NPV, negative predictive value*.

**Figure 6:**
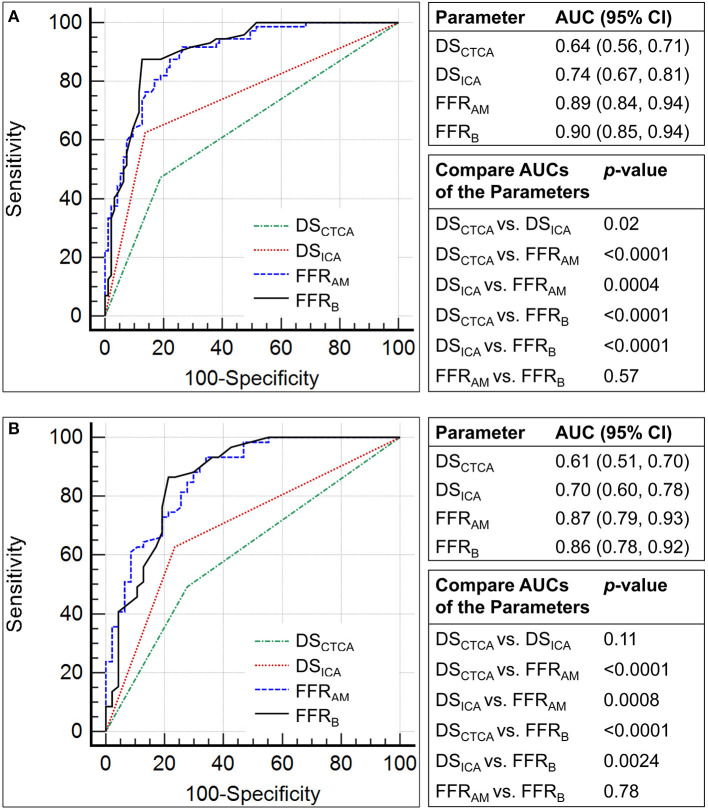
Comparison of the receiver operating characteristic curves for the discrimination of myocardial ischemia (fractional flow reserve, FFR ≤0.8) using diameter stenosis (DS; DS_CTCA_ and DS_ICA_, with a threshold of 70%) and non-invasive FFR (FFR_AM_ and FFR_B_) on **(A)** per-vessel and **(B)** per-patient levels.

The performance metrics using DS_CTCA_ ≥70%, DS_ICA_ ≥70%, FFR_AM_ ≤ 0.8, and FFR_B_ ≤ 0.8 to discriminate ischemic lesions are compared in Table 3 and Figure 6. On a per-vessel level, the ROC AUCs for FFR_AM_ and FFR_B_ were significantly higher than those for DS_CTCA_ [0.64 (0.56, 0.71)] and DS_ICA_ [0.74 (0.67, 0.81)]. On a per-patient level, the ROC AUCs for FFR_AM_ and FFR_B_ were significantly higher than those for DS_CTCA_ [0.61 (0.51, 0.70)] and DS_ICA_ [0.70 (0.60, 0.78)].

With invasive FFR as a reference standard, 32 lesions in 27 patients were wrongly classified with FFR_AM_ and 22 lesions in 19 patients were wrongly classified with FFR_B_. At both per-vessel and per-patient levels, FFR_AM_ and FFR_B_ achieved a significantly improved accuracy compared with DS_CTCA_ ≥50%, DS_ICA_ ≥50%, DS_CTCA_ ≥70%, and DS_ICA_ ≥70% (Table 3).

### Computational Time FFR_AM_ vs. FFR_B_

Excluding image acquisition and segmentation, the computational time for FFR_B_ was 48 ± 36 min (range 0.12 to 3.67 h) using parallel computation on a Dell T7800 workstation. The corresponding computational time for FFR_AM_ was 2.2 ± 0.9 min, using a single CPU of the same workstation.

## Discussion

In this study, we have developed a novel analytical method to determine FFR_AM_ non-invasively from patient-specific 3D models reconstructed from CTCA images. The FFR_AM_ exhibited a good correlation with invasive FFR and had a diagnostic performance close to CFD-based FFR_B_. We have also demonstrated the diagnostic performance of FFR_B_ in a prospective study design. The computational time for FFR_AM_ was much shorter than that for FFR_B_.

Our analytical model compartmentalized the stenosed coronary vessel into segments with distinct geometry to simplify the calculation of the corresponding pressure drops. We used anatomical information and LVM to calculate the flow rate through lesions and then input them into empirical equations with anatomical parameters measured on 3D coronary models to calculate energy loss due to the expansion and constriction of the lumen cross-section, which facilitates non-invasive FFR_AM_ calculation. A major advantage of estimating FFR_AM_ non-invasively using the analytical model is computational speed since the computational cost of CFD is eliminated. The analysis took slightly more than 2 min on a single CPU. This speed was achieved with little compromise in diagnostic accuracy. The flow rates through the lesions calculated in our analytical method using only anatomical information had a good correlation with that obtained by CFD simulation (*r* = 0.95), and the derived FFR_AM_ demonstrated a fair correlation and good agreement with invasive FFR and was close to FFR_B_. For the diagnosis of ischemia, FFR_AM_ had similar AUC (0.89 *vs*. 0.90, *p* = 0.57 and 0.87 vs. 0.86, *p* = 0.78 on per-vessel and per-patient bases, respectively) and specificity (86 *vs*. 86% and 79 vs. 77% on per-vessel and per-patient bases, respectively) but with slightly lower sensitivity (75 vs. 88% and 73 vs. 86% on per-vessel and per-patient bases, respectively) and NPV (82 vs. 90% and 70 vs. 82% on per-vessel and per-patient bases, respectively) compared with FFR_B_. Notably, both methods had superior diagnostic performance to routine methods, including DS_CTCA_ and DS_ICA_.

The FFR_AM_ derived from the lesion lumen area, length, flow entry and exit angles, and flow rate with fluid equations is different from diameter stenosis and other measurements of coronary morphologic information. It is related more to coronary hemodynamics and physiology. The lesion length and diameter have been employed by other investigators as indirect measures of fractional flow reserve (29). Our current study showed a greater mean value of lesion length (11.46 ± 7.40 vs. 10.07 ± 5.97 mm, *p* = 0.223) and a smaller lesion area (3.05 ± 1.56 vs. 4.56 ± 2.41 mm^2^, *p* < 0.0001) in the group with FFR ≤ 0.8 vs. the group with FFR >0.8. As a result, the estimated coronary morphologic index [eg., lesion length/minimal lesion diameter (29)] from our study is significant greater (12.3 vs. 4.8, *p* < 0.0001) in the group with FFR ≤ 0.8 vs. the group with FFR >0.8, which is in agreement with the findings from the study of Li (29). In addition to the aforementioned coronary morphologic index, other lesion geometric parameters, like flow entry and exit angles to lesions, have been associated with fluid convective and diffusive energy loss and pressure drop (3, 4). We have incorporated these additional elements in formulating the expressions for FFR_AM_ calculation. By decomposing a coronary lesion model of finite length into a spatial series of a proximal contracting segment, middle stenotic segment, and distal expanding segment to derive the model equations, FFR_AM_ presents an integrated assessment of coronary hemodynamics that provides a more accurate assessment of coronary physiology than morphologic stenosis index.

### CTCA-Based Non-invasive FFR to Discriminate Ischemic Lesions

Recent developments in CFD and CTCA imaging have made the calculation of non-invasive FFR feasible. NXT (6) and Discover-flow trials (5) employed standard transient CFD simulation and reported accuracy, sensitivity, specificity of 86, 84, and 86% (6) and 84.3, 87.9, and 82.2% (5), respectively, on a per-vessel basis and 80, 85, and 79% (6) and 87, 93, and 82% (5) on a per-patient basis. In the current study, our previously developed reduced-order CFD-based FFR_B_ (9) yielded commensurate accuracy, sensitivity, and specificity of 87, 88, and 86% on a per-vessel basis and 82, 86, and 77% on a per-patient basis. While there are limitations to cross-trial comparisons, the AUCs of FFR_B_ [0.90 (0.85, 0.94) and 0.86 (0.78, 0.92) on per-vessel and per-patient bases, respectively] and FFR_AM_ [0.89 (0.84, 0.94) and 0.87 (0.79, 0.93) on per-vessel and per-patient bases respectively] were in the similar range of and were intermediate between the AUCs reported for FFR_CT_ in the DeFACTO [0.79 (0.72, 0.87) on a per-patient basis] (30) and NXT trials [0.93 (0.91, 0.95) and 0.90 (95% CI: 0.87 to 0.94) on per-vessel and per-patient bases, respectively) (6), suggesting that both compared favorably with standard transient CFD-based approaches.

While CFD-based non-invasive FFR can improve the diagnostic performance of DS_CTCA_ alone, it is provided as a remote service with a long turnaround time due to the significant computational costs incurred for mesh generation and iterative solutions to solve numerical equations, which are procedures intrinsic to flow simulation (5, 6). To facilitate on-site non-invasive FFR computation, Coenen *et al*. (31) modeled the coronary vessel as a 1D segment for simulation and mapped the calculated cFFR onto the 3D model reconstructed from CTCA images. The computational time was reduced to 5–10 min per patient, but the accuracy was only 74.6% with invasive FFR as reference (31). Machine-learning based artificial intelligence (AI) algorithms were introduced to reduce the calculation time of non-invasive FFR in some studies that were mainly based on retrospective investigations (13–15). These required ample synthetic datasets for training before the AI algorithms could be applied. Another option to reduce computational time entails the use of analytical models. Huo *et al*. (16) reported an analytical method to estimate FFR from the dimensions of stenosis and hyperemic coronary flow. The method relied on *in vitro* or animal experiments to obtain hyperemic coronary flow, which hindered its applicability outside the laboratory. In contrast, our new analytical model uses only anatomical information and does not require *in vitro* or *in vivo* experiments. With relatively similar diagnostic performance as and lower computational demand than CFD-based approaches, the application of FFR_AM_ for on-site non-invasive FFR analysis may become feasible.

### Linkage of Parameters in the Analytical Model to Features in AI Algorithms

AI algorithms can facilitate non-invasive FFR estimation (13). The judicious selection of input parameters plays an important role in the accuracy of machine learning. Table 2 shows the list of anatomical features measured on or derived from CTCA-derived 3D coronary models and their discriminative capability for ischemic lesions. These parameters can aid in the feature selection of diagnostic AI algorithms. Flow quantitation by machine learning can also be facilitated using anatomical features since the coronary flow rates in the lesions that were derived from anatomical information showed a strong correlation with the CFD simulation results in our study (*r* = 0.95, *p* < 0.0001).

Minimal lumen area measured on intravascular ultrasound has been correlated with FFR-ascertained ischemia (32), and a minimal lumen area ≤ 3.0 mm (2) indicates a high likelihood of significant obstruction in a normal-sized coronary vessel (32). Accordingly, minimal lumen area has been adopted as one of the features for angiography-based machine learning algorithms (33). In our study, the lumen area at the site of maximum stenosis (*A*_s_) was significant smaller in ischemic *vs*. non-ischemic lesions (3.05 ± 1.56 vs. 4.56 ± 2.41 mm^2^, *p* < 0.0001), and we believe that it is a prime candidate for feature selection in machine learning. Due to curvature changes in the stenotic region, the flow entrance and exit angles α and β were significantly different between the ischemic and non-ischemic lesions in this study. As such, their effects on FFR prediction can be explored in future machine learning, together with other anatomical parameters, such as lumen areas, lesion lengths, *etc*.

Despite the potential of AI to non-invasive FFR, its clinical application remains challenging. The problem in AI lies in training data paucity, clinical interpretation, commercial deployment, and safety. Our method is based on coronary morphologic parameters and fluid dynamic principles and does not need training data. Importantly, the calculation can be completed with a much shorter computational time than full computational fluid dynamics. Lastly, we have developed a visualization system for physicians to view the computational results from both anatomic modeling and calculated FFR_AM_ and FFR_B_. This holds a potential application for the further personalized management of CAD patients like virtual stent simulation in our recent publication (34).

### Limitations of the Study

There are limitations in this study. First, a high calcium score may preclude accurate segmentation, which is a problem common to all CTCA-based analysis. The lumen segmentations were carefully examined by two experienced radiologists in the current study to ensure the accuracy of the results. Second, hyperemia was induced by either an intravenous infusion or intracoronary bolus of adenosine; nonetheless, prior studies have reported that the intravenous infusion of adenosine yielded an identical FFR result compared with intracoronary bolus (35). Lastly, this study did not use recently developed instantaneous wave-free ratio and resting full-cycle ratio non-hyperemic indexes of coronary artery stenosis severity as a reference method.

## Conclusions

In this prospective multicenter study, an analytical method that calculates non-invasive FFR_AM_ from CTCA and anatomical features offers a novel and expeditious non-CFD approach that demonstrated good diagnostic performance for detecting ischemic coronary lesions as ascertained by invasive FFR.

## Data Availability Statement

The original contributions presented in the study are included in the article/Supplementary Material, further inquiries can be directed to the corresponding authors.

## Ethics Statement

The studies involving human participants were reviewed and approved by SingHealth Centralised Institutional Review Board. The patients/participants provided their written informed consent to participate in this study.

## Author Contributions

STL and LZ conceived the study design. J-MZ and HH analyzed the data. R-ST, PC, JF, LT, CC, CO, WH, LB, GK, AL, MC, KC, PL, AW, SYT, TC, STL, and LZ interpreted the results. JA performed the statistical analysis. J-MZ drafted the manuscript. R-ST, PC, JF, LT, CC, CO, RL, GC, SL, WH, JA, LB, GK, AL, MC, KC, PL, AW, SYT, TC, STL, and LZ edited and revised the manuscript. All authors read and approved the final manuscript.

## Funding

This study has received funding from the National Medical Research Council Singapore (NMRC/BnB/0017/2015 and MOH-000358). The funder had no role in the design and conduct of the study; collection, management, analysis, and interpretation of the data; and preparation, review, or approval of the manuscript.

## Conflict of Interest

The authors declare that the research was conducted in the absence of any commercial or financial relationships that could be construed as a potential conflict of interest.

## Publisher's Note

All claims expressed in this article are solely those of the authors and do not necessarily represent those of their affiliated organizations, or those of the publisher, the editors and the reviewers. Any product that may be evaluated in this article, or claim that may be made by its manufacturer, is not guaranteed or endorsed by the publisher.

## Abbreviations

AM: analytical method
AUC: area under the receiver operating characteristic curve
CAD: coronary artery disease
CFD: computational fluid dynamics
CTCA: computed tomography coronary angiography
DS: diameter stenosis
FFR: fractional flow reserve
ICA: invasive coronary angiography
L: lesion length
LAD: left anterior descending
LR: likelihood ratio
NPV: negative predictive value
PPV: positive predictive value
Q: flow rate
ROC: receiver operating characteristic
SD: standard deviation
α: flow entrance angle
β: flow exit angle.
