# Supplementary material for: Diagnostic Performance of Fractional Flow Reserve From CT Coronary Angiography With Analytical Method
Source: Front Cardiovasc Med. 2021 Oct 20;8:739633. doi: 10.3389/fcvm.2021.739633 (PMC8564016; doi:10.3389/fcvm.2021.739633)
Supplement: Supplementary file 1 [file Data_Sheet_1.DOCX]

The Mathematical equations used for assessing the calculated FFR from CTCA

**Pressure wires/catheters used for measuring invasive FFR**

Invasive FFR was measured using one of following pressure wires/catheters: PressureWire X (Abbott Vascular, Illinois, USA), PressureWire Aeris (St Jude Medical, Minnesota, USA), Volcano Verrata pressure guidewire (Philips, Amsterdam, Netherlands), Navvus microcatheter (ACIST, Minnesota, USA), Comet Pressure guidewire (Boston Scientific, Massachusetts, USA).

**CTCA image segmentation and 3D model reconstruction**

CTCA images were loaded to QAngio CT software (version 3.0, Medis) for offline 3D reconstruction and coronary artery segmentation. Coronary ostia were identified from the reconstructed 3D whole-heart volume and centerlines of coronary arteries and approximate lumen outlines automatically traced using a fast vessel tracking algorithm. From curved multiplanar reformatted longitudinal and corresponding orthogonal cross-sectional views, lumen borders of coronary artery branches with lumen diameters ≥1.5 mm were further delineated using a semi-automatic algorithm with manual adjustments if necessary [1].

**Computation of non-invasive FFR_AM_ with analytical model**

In our analytical model, ${FFR}_{AM}=1-\frac{\Delta P_{1}+\Delta P_{2}}{P_{a}}$, where *P_a_* is patient-specific mean aortic pressure at hyperemia, estimated as mean cuff pressure minus 6.8 mmHg to account for pressure drop during hyperemia [2]; and ∆*P*_1_ and ∆*P*_2_, pressure drops across the coronary lesion and from the coronary orifice to the proximal end of the coronary lesion, respectively.

The latter is calculated from Hagen–Poiseuille equation $\Delta P_{2}=\sum\frac{8\pi\mu L_{i}Q_{i}}{A_{i}^{2}}$, where *μ* represents a constant viscosity of the blood (*μ=*0.0045 Pa·s); and *A_i_*, *L_i_* and *Q_i_*, lumen area, length, and flow rate of the *i*-th coronary branch of the left or right coronary tree, respectively.

By law of energy conservation, ∆*P*_1_ entails convective and diffusive energy losses as well as energy loss attributable to sudden constriction and expansion [3]. Flow separation and swirling that exacerbate energy losses and pressure drops are related to features such as lesion length, lumen area, flow entrance and exit angles etc. [4]. We applied these considerations in series to a coronary lesion model of total length *L* decomposed schematically into three components: a proximal contracting segment of length *L*_ps_ and distal expanding segment of length *L*_sd_, which bookend a middle maximally stenosed segment of finite length *L*-*L*_ps_-*L*_sd_, (**Supplementary Figure 1**). Respective pressure drops across the three segments Δ*P*_ps_, Δ*P*_sd_ and Δ*P*_ss_ sum up to ∆*P*_1_ and are, from a mechanical engineering perspective, analogous to pressure drops across contracting, expanding, and straight pipes, respectively. **Figure 3 (f)** illustrates how we measured anatomical parameters *L*, *L*_ps_, and *L*_sd_, as well as *A*_P_, *A*_d_, *A*_s_, the lumen areas at the proximal and distal ends of the coronary lesion and the maximally stenosed segment, respectively. From these parameters, flow entrance (*α*) and exit (*β*) angles were derived to facilitate calculation of Δ*P*_ps_ and Δ*P*_sd_ as below.

The flow entrance angle *α* at the interface of the proximal contracting passage [4] and the maximally stenosed segment (**Supplementary Figure 2**) is calculated as:

$\alpha=2\times Atan\left( \frac{\sqrt{\frac{A_{P}}{\pi}}-\sqrt{\frac{A_{s}}{\pi}}}{L_{ps}} \right)$ (A-1)

where *L*_ps_ is measured on 3D models as the length between the proximal ends of the coronary lesion and its maximally stenotic segment, which have lumen area A_p_ and A_s_, respectively [4]. The pressure drop ∆*P*_ps_ along the proximal contracting passage has a pressure loss coefficient *δ*_ps_:

$\delta_{ps}=k_{1}\times k_{2}$ (A-2)

where *k*_1_ and *k*_2_ are related to *α* and lumen area ratio *r* (=*A*_s_/*A*_p_), respectively. Through polynomial curve fitting, these relationships can be represented as [4]:

$k_{1}=0.52493-0.01656\times\alpha+2.35\times{10}^{-4}\times\alpha^{2}-8.04\times{10}^{-7}\times\alpha^{3}$ (A-3)

$k_{2}=0.41126-0.1244\times r-0.08625\times r^{2}-0.19425\times r^{3}$ (A-4)

Accordingly, ∆*P*_ps_ is calculated as:

$\Delta P_{ps}=\frac{\rho{Q_{AM}}^{2}}{2}\left[ \frac{1+\delta_{PS}}{{A_{s}}^{2}}-\frac{1}{{A_{P}}^{2}} \right]$ (A-5)

where *ρ* and *Q_AM_* represent blood density and hyperemia flow rate through the lesion, respectively.

Similarly, the flow exit angle *β* at the interface of the maximally stenosed segment and the distal expanding segment is calculated as:

$\beta=2\times Atan\left( \frac{\sqrt{\frac{A_{d}}{\pi}}-\sqrt{\frac{A_{s}}{\pi}}}{L_{sd}} \right)$ (A-6)

where *L*_sd_ is measured on 3D models as the length between the distal ends of the maximally stenosed segment and the coronary lesion, which have lumen area *A*_s_ and *A*_p_, respectively. Flow separation and swirling as the lumen area increases induce a pressure drop ∆*P*_sd_ along the distal expanding segment that has a pressure loss coefficient *δ*_sd_ given by:

$\delta_{sd}=k_{3}\left( \frac{A_{d}}{As}-1 \right)^{2}$ (A-7)

where, *k_3_*, through polynomial curve fitting, can be represented as [4]:

$k_{3}=0.0164\times\beta-5\times{10}^{-5}\times\beta^{2}+2\times{10}^{-5}\times\beta^{3}$ (A-8)

Accordingly, ∆*P*_sd_ is calculated as:

$\Delta P_{sd}=\frac{\rho{Q_{AM}}^{2}}{2}\left[ \frac{1}{{A_{s}}^{2}}+\frac{\delta_{sd}-1}{{A_{d}}^{2}} \right]$ (A-9)

Both Hagen–Poiseuille equations and Eq. (A-5) as well as Eq. (A-9) required the hyperemic flow rate through the coronary branches for calculation. We first calculated resting flow rate through the coronary vessel using the scaling law [5]:

$Q_{i}=Q_{inlet}\times\frac{D_{i}^{7/3}}{\sum_{j=1}^{N} D_{j}^{7/3}}$ (A-10)

where *Q_i_* is flow rate in the *i*-th branch vessel; *Q_inlet_* , flow rate at orifice of left or right coronary artery tree; and *D_i_*, mean lumen diameter of the *i*-th branch of left or right coronary artery tree with *N* branches. *Q_inlet_* was calculated from total coronary flow rate and left and right coronary artery flow distribution determined by CTCA-assessed LVM [6] and scaling law [5], respectively.

Hyperemic flow rate *Q*_AM_ through a coronary lesion located at the *i*-th branch of the coronary artery tree was then computed as *kQ_i._* [3]*.* The coefficient *k* reflects the magnitude of flow increase at hyperemia and is dependent on the diameter stenosis of the lesion (*DS*):

$k=4.7-1.8\times DS+5.6{\times DS}^{2}-8.5\times{DS}^{3}$ (A-11)

Here $DS=1-\sqrt{\frac{A_{s}}{\pi R_{ref}^{2}}}$ and reference diameter

$R_{ref}=\sqrt{\frac{A_{d}}{\pi}}+\left( \sqrt{\frac{A_{p}}{\pi}}-\sqrt{\frac{A_{d}}{\pi}} \right)\frac{L_{sd}+L-L_{ps}}{2L} \left( if A_{p}\geq A_{d} \right)$ (A-12)

$R_{ref}=\sqrt{\frac{A_{p}}{\pi}}+\left( \sqrt{\frac{A_{d}}{\pi}}-\sqrt{\frac{A_{p}}{\pi}} \right)\frac{L_{ps}+L-L_{sd}}{2L} \left( if A_{p}<A_{d} \right)$ (A-13)

Inputting *Q*_AM_ to the Hagen–Poiseuille equations and Eq. (A-5) as well as Eq. (A-9), noninvasive FFR_AM_ is computed without the need for CFD simulation as

${FFR}_{AM}=1-\frac{\Delta P_{ps}+\Delta P_{sd}+\Delta P_{ss}+\Delta P_{2}}{Pa}$ (A-14)

∆*P*_2_ is the pressure drop between the coronary orifice and the proximal end of the coronary lesion; and ∆*P*_ss_, pressure drop along the straight maximally stenosed segment of the lesion with constant area *A*_s_ and finite length (*L*-*L*_ps_-*L*_sd_). Both ∆*P*_2_ and ∆*P*_ss_ can be calculated using the Hagen–Poiseuille equation. *P_a_* is subject-specific mean aortic pressure at hyperemia.

**Supplementary Figure 2** shows an example of how to select the proximal “P” and distal ends “D” of the coronary artery lesion as well as the maximally stenosed point “S” at which measure lumen area and length parameters. Coronary lumen area along the stenosed coronary artery derived from 3D curved multiplanar reformatting of the coronary artery along a centerline are plotted against the straightened length of the artery from proximal (left) to distal (right). The curve is smoothed using a median or other low-pass filter to minimize the effect of noise. Coronary stenosis is maximal at point “S”, which corresponds to the minimum lumen area. While depicted as a point here, “S” is in reality of the finite length (**Supplementary Figure 1**). “P” and “D” correspond to proximate points on plotted curves with maximal absolute values of either curvature or change of slope to the left and right of point “S”, respectively (**Supplementary Figure 2**). Curvature is defined as the reciprocal of the radius of an osculating circle and is computed using the independent coordinates method for planar curves [7]. Change of slope is calculated as the difference of slopes for two adjacent points along the curve.

**Computation of non-invasive FFR_B_ based on reduced-order CFD simulation**

CFD simulation was performed on the reconstructed 3D coronary artery tree model assuming steady-flow with appropriate boundary conditions [8-10]. First, the computational domain was discretized to tetrahedral meshes using ANSYS Meshing (version 18, ANSYS, Inc.). Refined meshes were generated near the walls to ensure adequate flow resolution at the boundary layers. Approximately 0.5-1.2 million cells were generated to obtain mesh-independent results.

Under hyperemic conditions, total coronary ﬂow was estimated at 4.76 times resting flow (computed from left ventricular mass as above) as coronary resistance reduced to 0.21 of resting value [2], and distributed between the left and right coronary artery trees according to the scaling law [11]. To facilitate solving governing equations for CFD simulation, total pressure boundary conditions were assigned to the inlet based on subject-specific mean aortic pressure *P_a_* [2] and aforementioned hyperemic inlet flow rates. Novel resistance and non-slip boundary conditions were assigned to outlets and wall, respectively. The convergence criterion was set at residual values ≤10^-4^. Simulation was performed using FLUENT (version 18, ANSYS, Inc.).

**
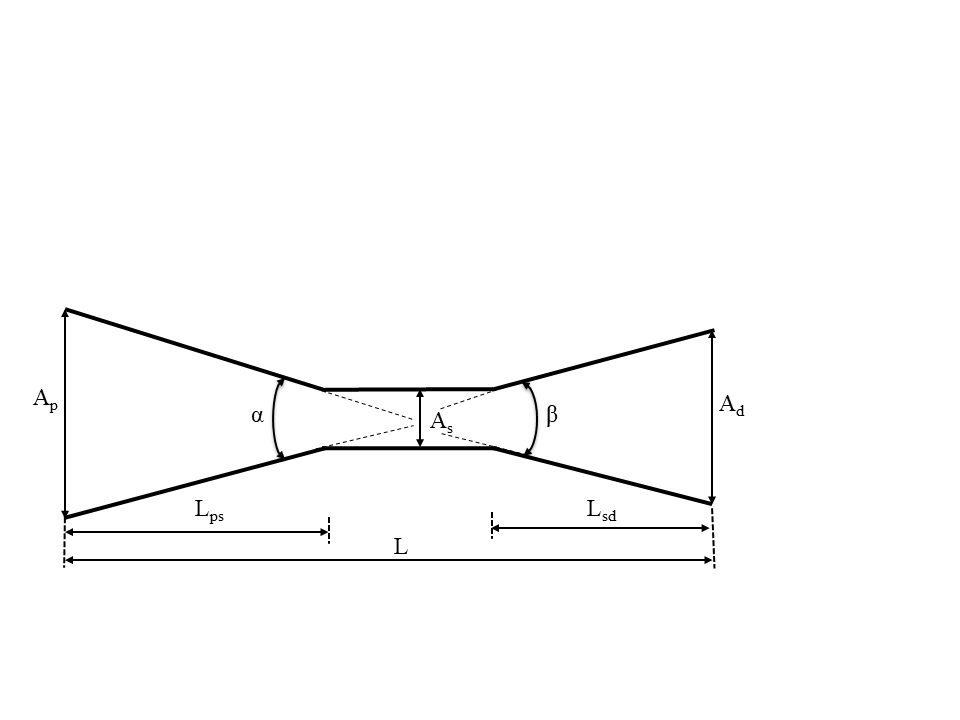
**

**Supplementary Figure 1.** Schematic drawing of the analytical model of a coronary lesion of total length L composed of a proximal segment of length L_ps_ with lumen area A_p_ that contracts to a maximally stenosed area A_s_, distal segment of length L_sd_ with lumen area A_s_ that expands to a distal area A_d_; and a middle straight segment of maximum stenosis bookended by aforementioned segments with constant area A_s_ and finite length L-L_ps_-L_sd_. *α* and *β* are flow entrance and exit angles when lumen area contracts from A_p_ to A_s_ and expands from A_s_ to A_d_, respectively.


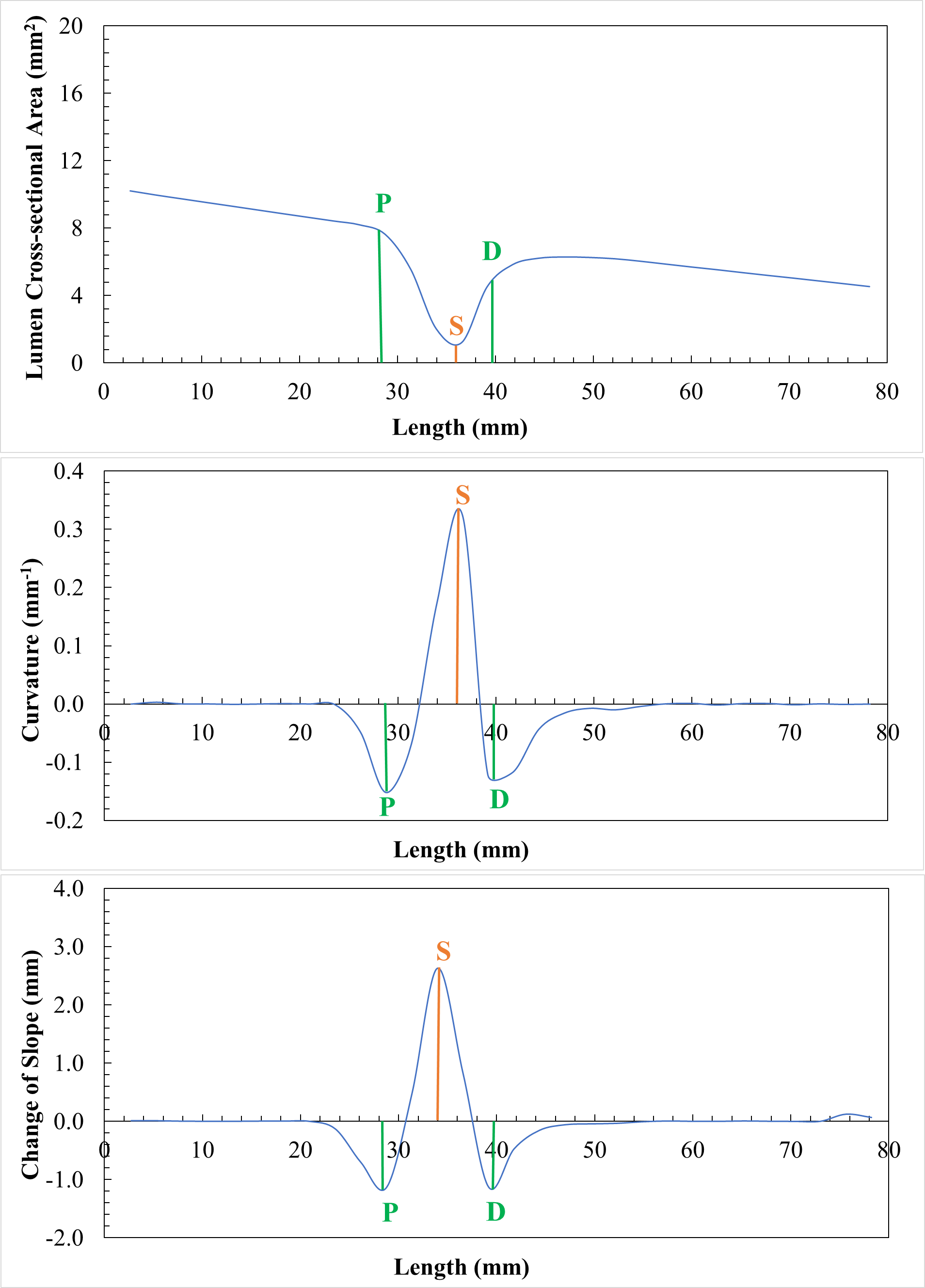


**Supplementary Figure 2.** The proximal “P” and distal ends “D” of the coronary artery lesion as well as the maximally stenosed point “S” are identified as inflection points on graphs of the lumen cross-section area (A) and the derived curvature (B) and change of slope (C) versus the reformatted straightened length of the coronary artery.

**References**

1. Heo R, Park HB, Lee BK, Shin S, Arsanjani R, Min JK, et al. Optimal boundary detection method and window settings for coronary atherosclerotic plaque volume analysis in coronary computed tomography angiography: comparison with intravascular ultrasound. Eur Radiol (2016) 26:3190-8. doi: 10.1007/s00330-015-4121-5
2. Wilson RF, Wyche K, Christensen BV, Zimmer S, Laxson DD. Effects of adenosine on human coronary arterial circulation. Circulation (1990) 82:1595–606. doi: 10.1161/01.cir.82.5.1595
3. Huo Y, Svendsen M, Choy JS, Zhang Z-D, Kassab GS. A validated predictive model of coronary fractional flow reserve. J R Soc Interface (2012) 9:1325–38. doi: 10.1098/rsif.2011.0605
4. Rennels D, Hudson H. Pipe Flow. New Jersey: John Wiley & Sons, Inc.; 2012.
5. Huo Y, Kassab GS. Intraspecific scaling laws of vascular trees. J R Soc Interface (2012) 9:190–200. doi: 10.1098/rsif.2011.0270
6. Wieneke H, von Birgelen C, Haude M, Eggebrecht H, Möhlenkamp S, Schmermund A, et al. Determinants of coronary blood flow in humans: quantification by intracoronary Doppler and ultrasound. J Appl Physiol (2005) 98:1076–82. doi: 10.1152/japplphysiol.00724.2004
7. Lewiner T, Gomes JD, Lopes H, Craizer M. Curvature and torsion estimators based on parametric curve fitting. Comput Graph (2005) 29:641–55. doi: 10.1016/j.cag.2005.08.004
8. Zhang JM, Zhong L, Luo T, Lomarda AM, Huo Y, Yap J, et al. Simplified models of non-invasive fractional flow reserve based on CT images. PLoS One (2016) 11:e0153070. doi: 10.1371/journal.pone.0153070
9. Zhang JM, Shuang D, Baskaran L, Wu W, Teo SK, Huang W, et al. Advanced analyses of computed tomography coronary angiography can help discriminate ischemic lesions. Int J Cardiol (2018) 267:208–14. doi: 10.1016/j.ijcard.2018.04.020
10. Zhong L, Zhang JM, Su B, Tan RS, Allen JC, Kassab GS. Application of patient-specific computational fluid dynamics in coronary and intra-cardiac flow simulations: Challenges and opportunities. Front Physiol (2018) 9:742. doi: 10.3389/fphys.2018.00742
11. Zhou Y, Kassab GS, Molloi S. On the design of the coronary arterial tree: a generalization of Murray’s law. Phys Med Biol (1999) 44:2929–45. doi: 10.1088/0031-9155/44/12/306
